# Novel participatory methods of involving patients in research: naming and branding a longitudinal cohort study, BRIGHTLIGHT

**DOI:** 10.1186/s12874-015-0014-1

**Published:** 2015-03-14

**Authors:** Rachel M Taylor, Jasjeet Mohain, Faith Gibson, Anita Solanki, Jeremy Whelan, Lorna A Fern

**Affiliations:** Cancer Clinical Trials Unit, University College London Hospitals NHS Foundation Trust, London, NW1 2PG UK; Pharmaceutical Advertising, London, HA5 2AA UK; Great Ormond Street Hospital for Children NHS Foundation Trust and London South Bank University, 103 Borough Road, London, SE1 OAA UK; National Institute for Health Research, University College London Hospitals Biomedical Research Centre, London, UK; Department of Oncology, National Cancer Research Institute Teenage and Young Clinical Studies Group, 1st Floor Central, 250 Euston Road, London, NW1 2PG UK

**Keywords:** Patient and public involvement, Participatory methods, Teenagers and young adults

## Abstract

**Background:**

Patient and public involvement (PPI) is central to research and service planning. Identifying effective, meaningful ways of involvement is challenging. The cohort study *‘Do specialist services for teenagers and young adults with cancer add value?’* follows young people for three years, examining outcomes associated with specialist care. Participant retention in longitudinal research can be problematic potentially jeopardising study completion. Maximising study awareness through high impact branding and publicity may improve study retention. Study names are typically generated by researchers rather than designed with patients.

We aimed to involve young people in developing a brand identity and name to ‘*Do specialist services for teenagers and young adults with cancer add value?’.*

**Methods:**

Nine young people aged 17–26 years diagnosed with cancer when aged 14–25 years participated in a one day workshop with further data collection at a patient conference. Methodology was similar to conventional branding and naming exercises and was divided into six stages. The workshop comprised five stages. Stage 1: ‘What’s in a brand’ allowed young people to enquire why brands/logos are important, Stage 2: ‘Brand Transformation’ identified what young people needed to know and believe about the study when approached about participation, Stage 3: ‘Brand Essence’ determined how we wanted the study to be perceived by young people, Stage 4: ‘What’s in a name’ identified potential names for the study. Stage 5: ‘Logo creation’ assembled the mood and feel of logos. Stage 6 was logo design and an electronic survey of 249 young people attending a patient conference.

**Results:**

BRIGHTLIGHT was the final study name and the brand essence (or study personality) was friendly, supportive and inspiring. Four logos were designed and the final logo received 47% (n = 115) of votes.

**Conclusions:**

Acceptance and retention to BRIGHTLIGHT is higher than anticipated (80% versus 60%), this may be related to our integral PPI strategy. We propose this reproducible methodology as an important, enjoyable, and novel way of involving patients in research and a welcome alternative to researcher-developed acronyms. Ideally this should be carried out prior to engaging with healthcare professionals to prevent confusion around study identity.

## Background

A challenge for longitudinal research is retention of study participants. Retention rates can be maximised by increasing awareness of studies through high impact branding and publicity campaigns [1]. Typically, academic-led research studies have a short title, often generated by the research team during protocol development. These are normally acronyms of the longer title perceived to be relevant and ‘catchy’ by the researchers. It is unclear whether study ‘branding’ is for professionals or patient benefit [2]. Research has shown that acronym-named studies recruit more quickly and are cited more frequently in research compared to studies without an acronym [3]. However, concern has been raised about the relevance and coercive nature of some study acronyms, for example HOPE and CURE [4].

Commercial companies invest considerable resource into naming and branding products. This typically involves consultation with the target audience for which a product is targeted. Involvement of target audiences or ‘consumer involvement’ is common in the commercial world and is increasingly considered to be an integral part of good research and service planning in healthcare [5]. Patient and public involvement [PPI] in research, refers to the active inclusion of patients, carers, service users, and stakeholders and may be defined as research being carried out ‘with’ or ‘by’ members of the public rather than ‘to’, ‘about’ or ‘for’ them [http://www.invo.org.uk/find-out-more/what-is-public-involvement-in-research-2/]. As a research team we have embedded the philosophy and practice of PPI into all of our research studies: using new learning to refine our approach to fully engage with the teenage and young adult [TYA] population.

*‘Do specialist services for Teenagers and Young Adults [TYA] add value?’* is a National Institute for Health Research funded programme of research [NIHR grant reference number RP-PG-1209-10013; www.brightlightstudy.com]. The core of the programme is a longitudinal cohort study determining outcomes associated with specialist TYA cancer services. The cohort study was preceded by a series of feasibility projects where we worked with young people as co-researchers [6,7]. In 2009, the feasibility work was named *‘The Essence of TYA care’* by the research team. As part of our retention strategy for the larger cohort study we proposed that young people taking part would receive regular study updates through a newsletter to keep participants up to date with study progress and this would be called ‘The Essence Echo’ in keeping with the theme of our feasibility work [8]. Nearing completion of our feasibility work, we held a one day workshop with our five young co-researchers and participants of the feasibility study, the purpose of this workshop was to disseminate the study results back to participants. During discussion at this workshop young people suggested both ‘*The Essence of TYA care’* and the ‘*Essence Echo’* were not suitable names and would fail to engage young people in the cohort study. We sought the views of a larger group of our target patient population who were attending the annual Teenage Cancer Trust patient conference – Find Your Sense of Tumour, 2011 [http://www.teenagecancertrust.org/get-clued-up/talk-to-other-young-people/find-your-sense-of-tumour/]. During the conference approximately 200 young people participated in an electronic survey following a short presentation about the proposed study (https://jtvcancersupport.com/2011/03/fysot-11-ncri-research-for-you/). We then asked young people what they thought of the name ‘Essence Echo’ for a study newsletter. Seven out of ten young people responded that the name ‘Essence Echo’ was either ‘pretty awful’ or ‘not that great’ with only a third citing it was a suitable title.

Recruitment and data collection from young people in the cohort study involves multiple agencies and is conducted over three years. Young people are screened, approached and recruited by healthcare professionals in their hospital up to four months after diagnosis. At five months they are contacted by an independent social research company (Ipsos MORI) to arrange participation in their first interview. After the first interview contact with young people is maintained by the main co-ordinating centre and data collection by Ipsos MORI. Thus the importance of an easily recognisable name was essential to link all three organisations to a single study over a prolonged period of time.

Amongst professionals the cohort study had become known as the ‘*The 2012 TYA Cancer Cohort Study’*. Shorter titles were proposed by members of the research team, for example, ‘Essential youth study’ or ‘colossal youth’. We informally tested these names with young people who did not have cancer attending a university and they were rejected. ‘Colossal youth’ in particular made young people think of an obesity study or stomas. This highlighted the emotive nature of study names particularly in a young population who are conscious of their own identity and are brand conscious.

Commercial companies aim to transform customer experience and brand engagement by activating and embedding customer voice into everything they do from product concept, to eventual launch and throughout the life span of the product. Using our philosophy of user involvement where young people have been integral throughout our feasibility work, we sought to engage with young people with cancer to establish an appropriate study name, logo and branding attributes prior to study opening, with guidance from independent creative advisors. Our aim was to involve young people in naming and branding the study in a way which was appealing to young people and which may then contribute to successful participation and retention.

## Methods

### Participants and setting

The study was undertaken during a one day workshop with subsequent data collection at a patient conference. Young people were recruited through Internet sites known to be used by young people with cancer, these included Teenage Cancer Trust Facebook page and https://jtvcancersupport.com/, invitations circulated to the main cancer treatment centres for young people in the United Kingdom. We also sent individual invitations to young people we had previously worked with.

Ten young people responded to the invitations. Of those, nine young people aged 17–26 years, eight female, who were diagnosed with cancer aged between 14–25 years, attended the workshop (details below). Participants worked in groups of three. Three researchers (LF, FG, RT) two independent creative advisors (JM, GP) and one young person (HM) who worked with us as a co-researcher also attended the workshop.

### Ethical considerations

Ethical approval was granted by a National Health Service Research Ethics Committee as part of our patient and public involvement strategy. Written consent for participation was obtained after scene setting during which young people were given more information about what the day would involve. Young people were free to leave at any time during the workshop. Young people were contacted individually by a researcher [LF] after the workshop to ensure the workshop content had not resulted in any emotional distress.

### Workshop format and procedures

**Figure 1:**
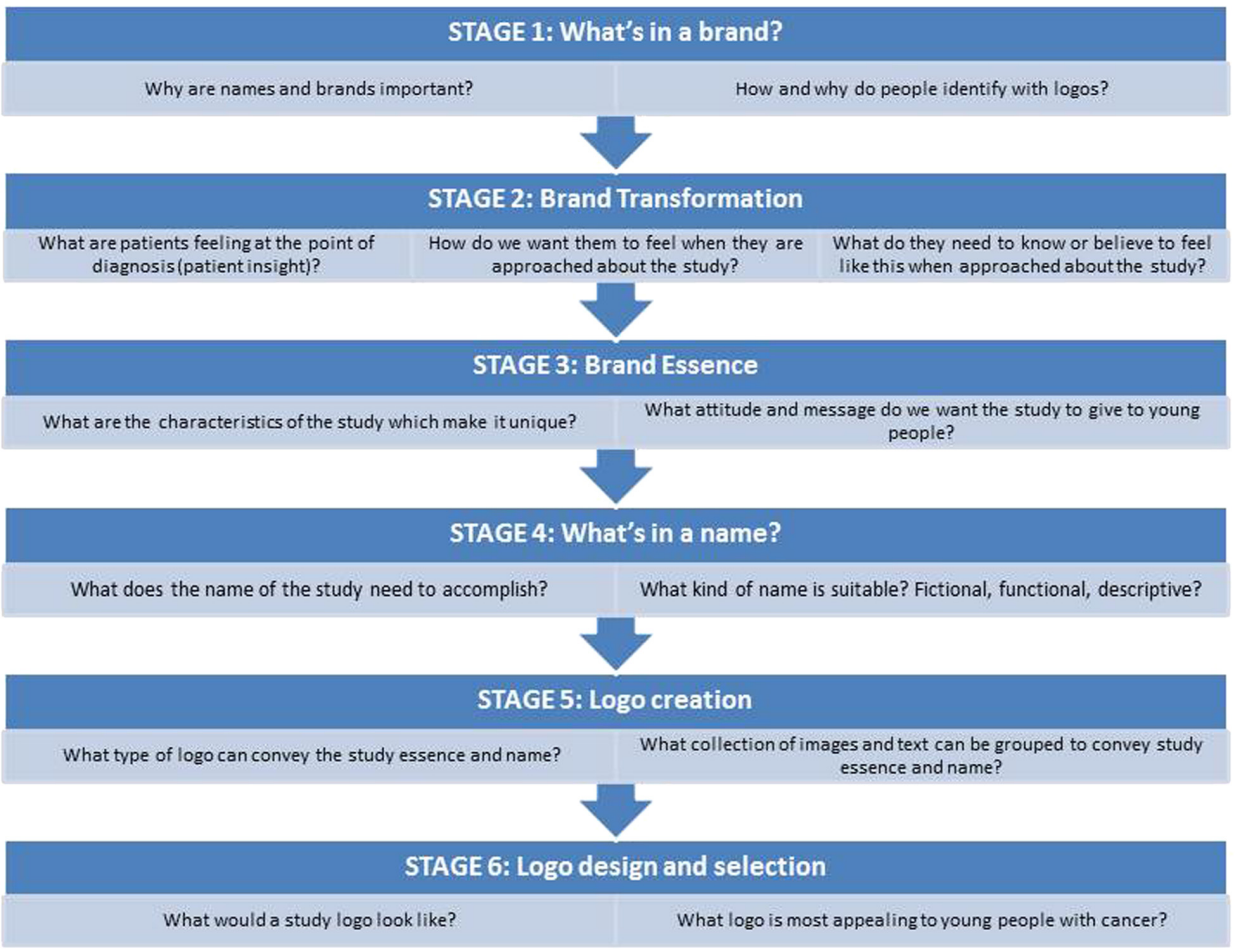
**Schematic diagram of the six stages used to name and brand the study.**

Participatory methods based on branding and naming methodology used by health care advertisers were employed [http://www.powerdecisions.com/branding-research.cfm#.Uwd2qRxZBVg]. The workshop, similar to that used to name and brand new products consisted of six stages designed to take the young people through the creative journey that commercial brands are subjected to [Figure 1]. The aim and methods of each stage are detailed below.

### STAGE 1: What’s in a brand?

#### Aim

To introduce the importance of branding, names, and logos to young people.

#### Methods

‘Logo and brand selection’.

Prior to young people arriving, the room was decorated with well known household logos relevant to young people. For example, Apple, North Face, Marmite, Skoda, Mercedes, BMW, Twitter and Facebook. Inspirational quotes were also placed on the wall to stimulate thought and creativity [Table 1]. A selection of branded and non-branded supermarket drinks and crisps were laid out [cola, lemonade, and water, flavoured and plain crisps]. Young people were asked to choose the logo from the wall they identified most with and to help themselves to a drink and snacks.

**Table 1:** **Illustrates examples of inspirational quotes placed on walls to stimulate thought and creativity during the workshop**

**Quote**	**Origin**
At this very moment, there are people only you can reach… and differences only you can make.	Mike Dooley
You’re happiest while you’re making the greatest contribution	Robert F. Kennedy
One person can make a difference and every person should try	John F Kennedy
Be the change you want to see in the world	Mahatma Gandhi
If you want things to be different perhaps the answer is to become different yourself	Norman Vincent Peale
Be a rainbow in someone’s cloud	Maya Angelou
If you light a lamp for someone else it will also brighten your path	Buddha

The creative advisors described the branding of ‘Apple’ as an example of design innovation, ease of use, simplicity and how this was reflected in all Apple advertising and communication. A short Apple video was shown [http://youtu.be/cpzvwkR1RYU]. The video was followed by simple explanations of some of terms and concepts to be used in the workshop. For example, ‘brand positioning’, is defined as how a product [in our case the study] is perceived in the minds of our customers [our patients] and what makes that product [our study] unique and beneficial. The brand is the impression created in the mind of the prospective consumer, or in this case potential study participants. To highlight the power of branding, participants were asked to identify whether they had selected branded or non branded crisps and drinks.

### STAGE 2: Brand transformation

#### Aim

To develop a brand transformation model, in order to identify the key attributes of the study which would make patients want to take in the study.

#### Methods

A ten minute presentation about the proposed study *‘Do specialist services for TYA add value?’* was given by a researcher [LF]. Young people were informed of the feasibility work and told that amongst professionals the study had become known as the ‘The 2012 TYA Cancer Cohort Study’. After this, there was an interactive discussion between the research team, young people and the creative advisors about the study. Study concept, design, recruitment methods, target population and outcomes were all considered.

Using the brand transformation model we gained patient insight into what are patients feeling and what is important to them at diagnosis, participants were asked to list how young people would be feeling at the point of diagnosis. We asked them to describe how they would want young people to feel when they are approached about the study and finally what young people needed to know about the study in order to move them from the feelings at diagnosis to wanting to take part in the study. Each group fed their answers back to the main group during an interactive discussion between participants, researchers and the creative advisors, this allowed illumination and clarification of points raised, particularly around emotions at diagnosis.

### STAGE 3: Brand essence

#### Aim

To determine the value of the study and how it might be perceived by young people (brand essence). The brand essence is the simplest possible statement of those characteristics that makes a brand unique and presents its future source of power, for our study we aimed for it to convey a voice, an attitude and a message to young people, the ‘take home’ message and feeling about the study we want young people to have when hearing about or participating in the study [9].

#### Methods

**Figure 2:**
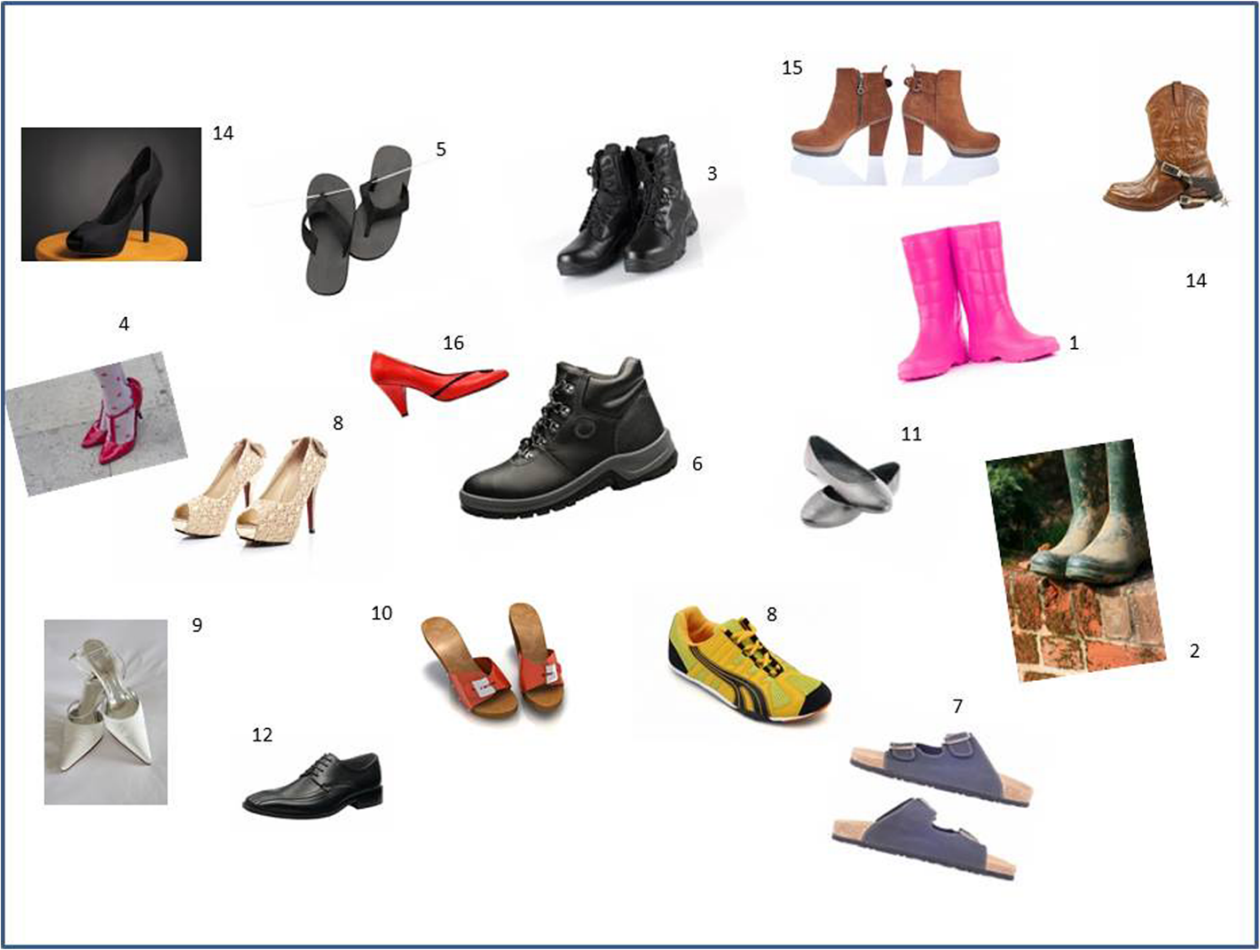
**Representation of footwear character board to determine brand identity.** If the study were footwear it would be a pink Wellington boot (readers can contact the authors for the original board used). Footnote for Figure 2: Copyrights for Figures. 1. Image by Phaitoon at FreeDigitalPhotos.net; 2. Image by Simon Howden at FreeDigitalPhotos.net; 3. Image by posterize at FreeDigitalPhotos.net; 4. Image by africa at FreeDigitalPhotos.net ; 5. Image by John Kasawa at FreeDigitalPhotos.net; 6. Image by John Kasawa at FreeDigitalPhotos.net 7. Image by artur84 at FreeDigitalPhotos.net; 8. Image by bigjom at FreeDigitalPhotos.net; 9. Image by Sharron Goodyear at FreeDigitalPhotos.net ; 11. Image by artur84 at FreeDigitalPhotos.net; 12. Image by John Kasawa at FreeDigitalPhotos.net; 13. Image by Boaz Yiftach at FreeDigitalPhotos.net; 14. Image by Gualberto107 at FreeDigitalPhotos.net; 15. Image by artur84 at FreeDigitalPhotos.net 16. Image by bigjom at FreeDigitalPhotos.net.

Each group was given a character board of a selection of cars, boats or shoes. Character boards are used in advertising to develop the brand personality or brand essence. Figure 2 illustrates a representation of the footwear character board (readers can contact the authors for the original board used). Each group chose which item on the board would best represent the study. The Groups fed back why they had chosen the item, which represented the study. Following group feedback and interactive discussion the creative advisors synthesised the results to assimilate the brand personality.

### STAGE 4: Name creation

#### Aim

To identify potential names for the study.

#### Methods

The creative advisors explained the importance of names and using drinks as examples illustrated fictional names such as Coco-Cola and Pepsi, functional names such as Vitamin Water or emotional names like Sunny Delight and Tropicana designed to associate the name with sunshine and happiness.

A researcher [LF] then showed examples of other study names and logos from current cancer studies. Examples included:

- ‘DISCOVERY’: a study looking at better pathways to cancer diagnosis in adult cancer patients, NIHR: RP-PG-0608-10045; http://discovery-programme.org/index.php. The ‘I’ in DISCOVERY is a lighthouse illuminating the rest of the word, and so the study name and logo is relevant for the study and was used an exemplar of an excellent study name and appropriate logo;
- EURAMOS-1: a treatment trial for osteosarcoma, the name was comprised of putting together the first few letters of words from the group who designed the study- **EUR**opean and **AM**erican **OS**teosarcoma Study Group, http://212.219.75.232/euramos/;
- DELAY: a qualitative study about young people’s diagnostic experiences;
- AsyMS: an **A**dvanced **Sy**mptom **M**anagement **S**ystem utilising mobile phone technology for patients to report cancer chemotherapy-related symptoms.

A recap of the results from STAGES 1–3 was given and young people worked in their groups to generate suitable names for the ‘2012 TYA Cancer Cohort Study’ taking into account the results from STAGES 1–3. Each group fed back their names and why they thought each one appropriate for the study, young people then worked individually and assigned two votes to their favourite name.

### STAGE 5: Logo creation

#### Aim

To generate guidance to direct creative designers to create appropriate logos.

#### Methods

The creative designers explained the concept of mood boards, which are typically used in the construct of logos. A range of logos were presented and interactive discussion about their meaning followed. Participants were given popular magazines and newspapers to browse through for ideas, glitter and a range of stationary. Each group constructed one mood board for the study names with the highest votes (BRIGHTLIGHT, LILAC and DROP) thus creating a visual tool to guide the logo designers for the overall of feel of the logo.

### STAGE 6: Logo design and selection

#### Aim

To create and select a study logo.

#### Methods

We involved young people in all stages of branding and naming the study apart from stage 6 logo design which required creative expertise in advertising and design. Following the workshop a number of logos were designed by a commercial logo design company and the creative advisors. Logos were sent to the nine young people who attended the workshop for feedback.

The final logo was selected by young people attending the annual patient conference ‘Find Your Sense of Tumour’ using previously described methodology [10]. A total of 249 young people aged 14–25 took part in the electronic survey, respondents were shown the four logos and asked to select which logo they thought was the most appealing.

## Results

The results of each stage of the logo development are described below in the six stages they were performed.

### Stage 1

Logo selection, young people had attributed both emotional and personal identity reasons for the logos they had selected. For example, ‘*This is the logo from my car, which my boyfriend bought me before he died*’, ‘*This is my computer logo which I love. It’s what I spent my wish on’*, *‘I am addicted to Twitter, my friends are always telling me not to tweet so much’*, ‘*I just love marmite’*.

Brand selection, of the nine participants, eight had chosen a branded drink and one had chosen supermarket cola; she explained she had picked it up by mistake but did not want to replace it as she had opened it before she realised. All nine had selected branded crisps. They spoke about their branded choices in terms of better quality, ‘*know what you’re getting’* and the image associated with supermarket brands compared to independent soft drinks and crisps.

### Stage 2

Table 2 lists the results of the brand transformation exercise. Patient insight at diagnosis, how the participants wanted patients to feel when they were approached about the study and what they needed to know and believe to facilitate this transformation from the initial feelings at diagnosis to wanting taking part in the study.

**Table 2:** **Brand transformation exercise, detailing patient insight, desired transformation and what they need to know/believe to achieve this**

**[A] Patient insight at diagnosis**	**[B] Desired transformation**	**[C]** ***Reasons to believe***
***How patients feel at diagnosis***	***How we want patients to feel when they hear about the study***	***What young people need to know in order to get from A to B***
Apprehensive	‘Want to be part of this!’	Aims and objectives of study
Apprehensive	Comforted	Approachable
Confused	Feel like their views are important	Approachable
Curious	Feel wanted – take part in national study	Communication – telephone, email etc.
Depressed	Feeling safe [details about them in study are confidential]	Getting answer
Eager	Helped	Groovy updates
Feelings at Diagnosis	Hope	Hope
Frustrated	Interested	How will it help?
Hope	Intrigued	Inclusive
Horror	Making a difference	Info about survival
In denial	Open	Inspired
Lonely	Part of the future	Longer term
Lost	Positive	Meet people like you
Not interested	Secure [non-isolated]	Not to be alone
Not interested [in study]	Sense of helpfulness	Notice boards
Outcast	Supported	Persuasive
Over loaded	To be driven	Point of contact
Over loaded	To be focussed	Regular newsletter
Over whelmed	To get involved	Simple
Pressured	Want to be part of it, want to make a difference	Something to look forward to
Hurt		Survival
Relieved [answer]		User friendly
Scared		Website
Shocked		Welcome packs
Unknown/uncertainty		What will it achieve?
Unlucky		You’re not on your own
Upset		
Why		

Duplicate entries are where more than one Group cited this emotion.

### Stage 3

The results of the brand essence are shown below.

- Group 1: If the study was a car it would be Knightrider: *Clever, unique, talks and is interactive, futuristic, popular, famous, nationwide, sleek design [work in progress], eye catching, making future better, first of its kind, everyone knows who the knight rider is, globally recognisable.*
- Group 2: If the study was footwear it would be a pink wellington boot [welly]: *Practical, stable, recognisable, foot wear is forever changing but a welly is reliable, fun, focus, simple, straight forward, evolving but no matter how much they change they are always originals with the same original focus.*
- Group 3: If the study was a boat it would be a cross between a life boat and an elegant yacht: *Part of a team, saves lives, goes through rough waters, feel safer. For the yacht element it looks nice, connects with the world, peaceful and stable.*

The final ‘brand essence’ or the personality of the study was friendly, supportive and inspiring for young people with cancer.

### Stage 4

A total of 29 names were proposed. Following individually voting five names made it to the final selection list [Table 3]. At the end of the workshop, through further group discussion, BRIGHTLIGHT was chosen as the final study name (reasons for exclusion of other four names detailed in Table 3).

**Table 3:** **The final list of proposed study names, the meaning behind each name and the reason for exclusion**

**Name**	**Definition**	**Reason for exclusion**
BRIGHTLIGHT	Light at the end of the tunnel, leading the way for other young people.	Not excluded
DROP	Each patient represents a drop of water; lots of drops make a ripple, which in turn make waves of change.	Too abstract
PICS	Powerful Innovate Cancer Study	Other existing studies with the same acronym
LILAC	Living in light accommodating cancer	Too feminine for mixed gender study
PYST	Powerful Youth Study	Inappropriate colloquialism

### Stage 5

**Figure 3:**
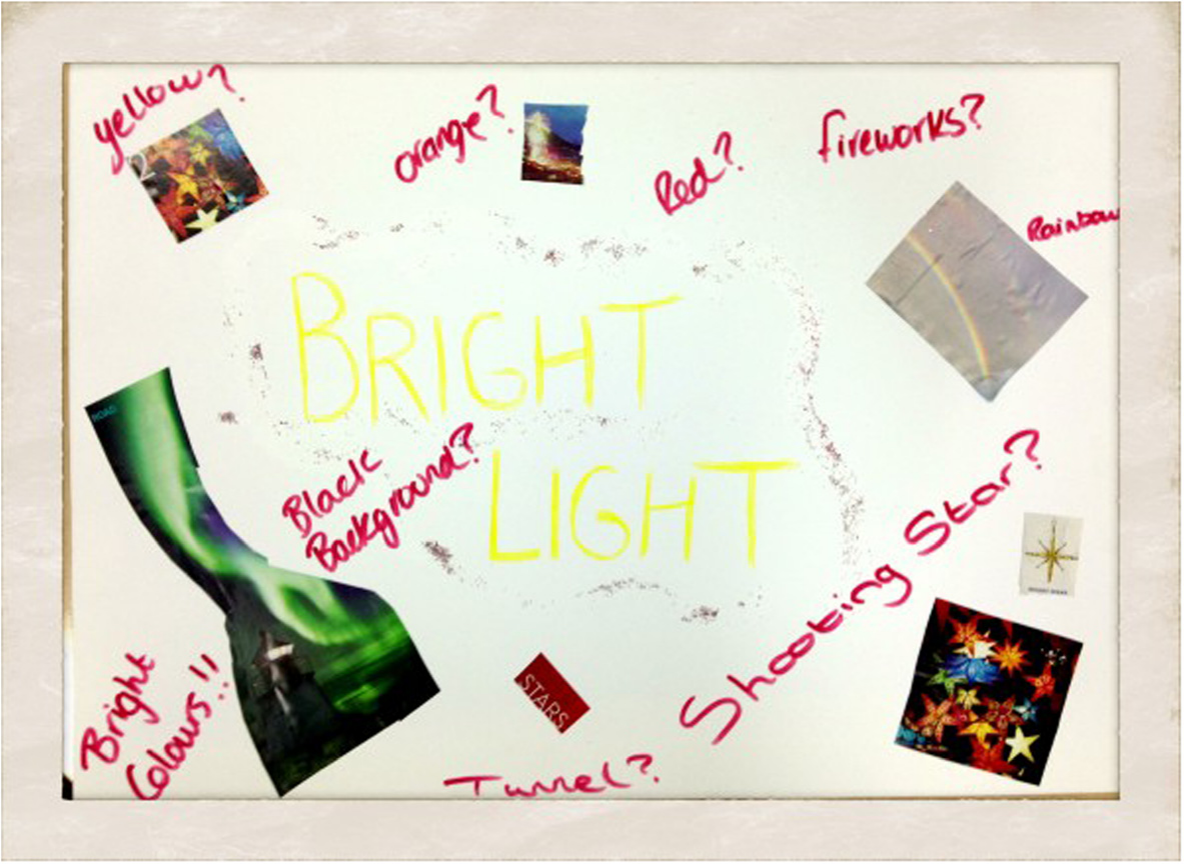
**BRIGHTLIGHT mood board used for the logo design.**

The BRIGHTLIGHT mood board is shown in Figure 3, this informed logo design.

### Stage 6

**Figure 4:**
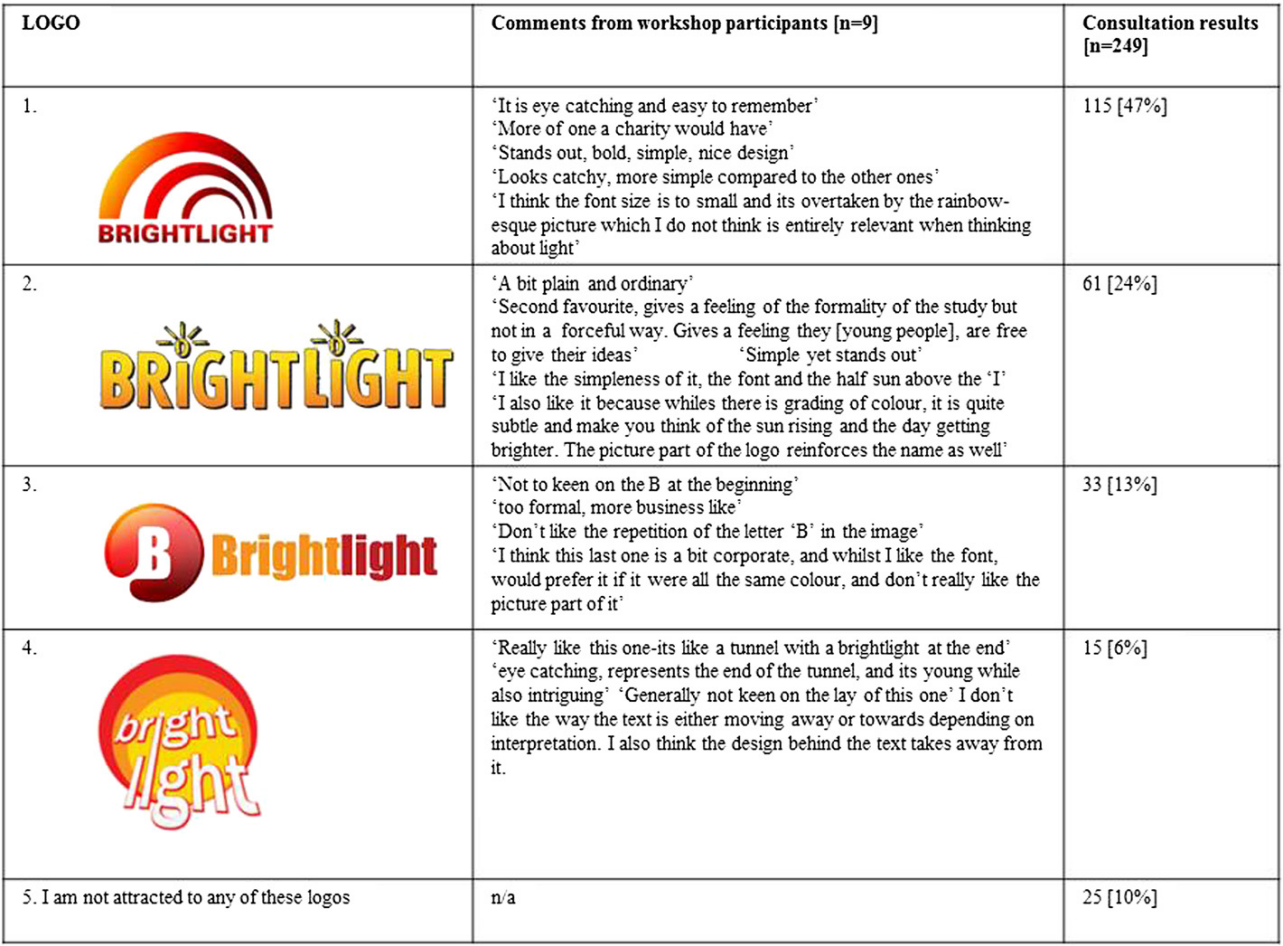
**The four final logos with comments on each from workshop participants and voting results from attendees at** ***‘Find Your Sense of Tumour, 2012’*** **.**

Four logos were selected for the final voting exercise. The results can be seen in Figure 4; logo number 1 was selected having received 47% of the votes [Figure 4].

### Potential impact

As with most user involvement initiatives the impact is not easily measurable. We are unable to measure what difference changing and branding the study name from ‘The 2012 TYA Cancer Cohort Study’ to BRIGHTLIGHT has made. However, despite lower than anticipated recruitment so far the refusal rate to BRIGHTLIGHT is less than 20%, versus an anticipated 35% which was based on refusal/consent rates in other published TYA cancer studies [11-13].

We can hypothesise that involving young people in study design, set-up and naming has positively influenced the acceptability of the study and therefore, for those who are approached about participation, acceptance rates are higher. Retention of participants for the second interview is approximately 80% versus an anticipated 60% and this may also be related to our PPI strategy. Anecdotally, young people and healthcare professionals responded favourably to the name change. In the process of re-branding the ‘2012 TYA Cancer Cohort Study’ we discovered the branding process is ideally undertaken at the point of study conception and not midpoint through gaining regulatory approval and increasing awareness of the study, however we recognise the financial constraints of doing this. A number of professionals failed to engage with the research team when materials started to bear the BRIGHTLIGHT logo as they did not realise this was the same as the ‘2012 TYA Cancer Cohort Study’, which they were already familiar with due to extensive publicity and awareness raising by the research team among the clinical and research community.

## Discussion

This paper describes the systematic way in which a research study *‘Do specialist services for TYA add value?’* was renamed, branded and assigned a suitable logo - BRIGHTLIGHT. In line with our patient and public involvement strategy, we strove to ensure that branding was directed by young people; this was especially important as attempts by the research team to rename the study had been largely rejected by young people. An appropriate and relevant study name may be of great value in promoting awareness of a study.

In commercial marketing the importance of the ‘brand’ is recognised; consumers develop a relationship with a product based on trust and the confidence they have in that particular brand [14]. The brand is the ‘conveyer of information’ and the ‘conveyer of image’ [15] and therefore defining the brand is fundamental to developing confidence and trust with a product. We sought to apply this to an academic study. We identified early in study development the importance of a suitable name, brand and presence for our study. A national cohort aiming to recruit young people with cancer shortly after diagnosis was anticipated to be ambitious. The study needed to be easily recognisable throughout the TYA cancer community, to both young people and health professionals, as a research project of value; that collaboration and participation in the study was something everyone aspired to. An important stage in this process was adopting a study name that would not only be instantaneously recognised but also respected.

There is little reference to the importance of naming and branding a study in health literature; however, in marketing there is a substantial body of evidence addressing the psychology behind branding and in developing methodology to ensure branding is undertaken in a rigorous way. Brands and branding allows the customers to differentiate one product from another with the use of name, design and advertising. This has an important effect on consumer preference and in fact a visual symbol can have greater value than words [16]. A successful brand starts with an effective product or service that consumers perceive as adding value. Added value equates to personal benefit and this needs to be sustainably and effectively communicated [17]. By working with young people throughout the feasibility work [6,7] and now in the naming and branding process we have ensured the study is relevant and of value to young people. On reflection, and our advice to other researchers would be to inform stakeholders about the pending name change and then engage in specific communication about the transition from the existing name to the new or in our case from ‘The 2012 TYA Cancer Cohort Study’ to ‘BRIGHTLIGHT’, ensuring that materials bore both names for a short period of time would also have been helpful.

Involving stakeholders as co-producers in branding products is becoming increasingly recognised [14]. In healthcare the importance of user involvement is embedded in clinical practice and good research. Our philosophy when branding BRIGHTLIGHT was that it must be directed by the consumers of our product or in this case, our study; under the guidance of experienced healthcare advertising executives and assisted by researchers, young people successfully re-branded ‘the 2012 Cohort Study’ as ‘BRIGHTLIGHT’. The name, logo and colour scheme selected by young people is easily recognisable on all study literature and our website [www.brightlightstudy.com].

## Conclusions

We have described an important step towards the success of BRIGHTLIGHT. Ensuring the name is identifiable, understandable and reputable is the building block to optimising recruitment and retention into the study. Rather than developing meaningless acronyms, we propose this as an important, enjoyable, reproducible and novel way of involving patients in research and engaging them from an early point in study design and set up.
